# Evolution of condensin and cohesin complexes driven by replacement of Kite by Hawk proteins

**DOI:** 10.1016/j.cub.2016.11.050

**Published:** 2017-01-09

**Authors:** Jonathan N. Wells, Thomas G. Gligoris, Kim A. Nasmyth, Joseph A. Marsh

**Affiliations:** 1 MRC Human Genetics Unit, Institute of Genetics and Molecular Medicine, University of Edinburgh, Edinburgh EH4 2XU, UK; 2 Department of Biochemistry, University of Oxford, Oxford, OX1 3QU, UK

## Abstract

Mitotic chromosome condensation, sister chromatid cohesion, and higher order folding of interphase chromatin are mediated by condensin and cohesin, eukaryotic members of the SMC (structural maintenance of chromosomes)–kleisin protein family. Other members facilitate chromosome segregation in bacteria [1]. A hallmark of these complexes is the binding of the two ends of a kleisin subunit to the apices of V-shaped Smc dimers, creating a tripartite ring capable of entrapping DNA (Figure 1A). In addition to creating rings, kleisins recruit regulatory subunits. One family of regulators, namely Kite dimers (Kleisin interacting winged-helix tandem elements), interact with Smc–kleisin rings from bacteria, archaea and the eukaryotic Smc5-6 complex, but not with either condensin or cohesin [2]. These instead possess proteins containing HEAT (Huntingtin/EF3/PP2A/Tor1) repeat domains whose origin and distribution have not yet been characterized. Using a combination of profile Hidden Markov Model (HMM)-based homology searches, network analysis and structural alignments, we identify a common origin for these regulators, for which we propose the name Hawks, i.e. HEAT proteins associated with kleisins.

## Teaser

Wells *et al.* show that a group of proteins that bind to and regulate the cohesin and condensin complexes share a common evolutionary origin, thus uniting these complexes.

## Main Text

**Figure 1:**
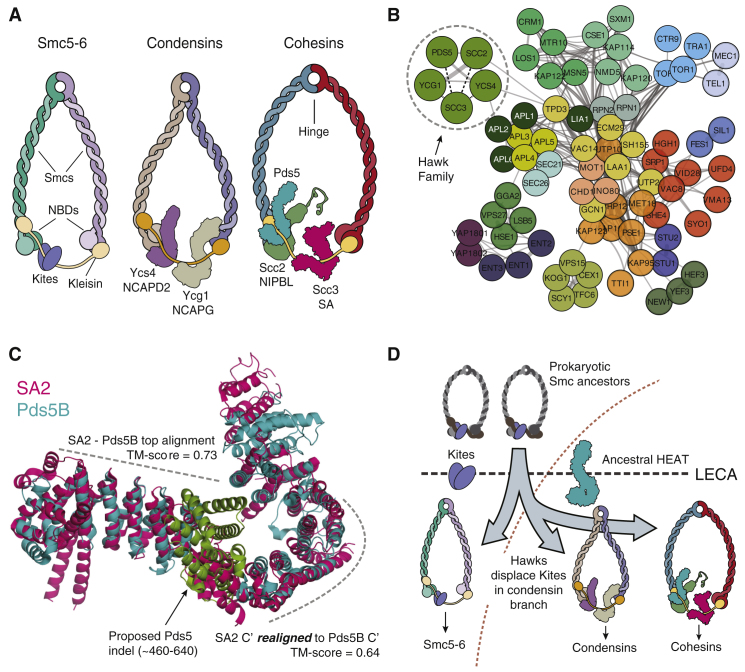
Hawk proteins form an evolutionarily related cluster and have displaced Kites in condensin and cohesin. (A) The amino- and carboxy-terminal domains of the Smc polypeptides together form the globular nucleotide-binding domain (NBD). Kleisin subunits (yellow) then close the ring, topologically entrapping DNA in the process. In Smc5-6, Kite proteins interact with kleisins. In cohesin, Scc2 competes with Pds5 for its binding site on the kleisin. (B) Cohesin (Scc3, Scc2, Pds5) and condensin (Ycs4, Ycg1) HEAT regulators form a compact Hawk cluster (circled). Each cluster represented by a single colour. For clarity, only edges with a mean probability ≥99.0% are shown. Disconnected sub-graphs are hidden — the exception to this is Scc3, which is the weakest member of the hawk cluster and has been manually added (dashed edges). Above this threshold, the Hawk cluster has strong links to TPD3 (Protein phosphatase PP2A regulatory subunit A) and APL2 (Clathrin assembly protein large beta-1 chain). Members of the latter family (white labels) retain some of the strongest links to both hawks and lokiarchaeal proteins. (C) Despite a pairwise sequence identity of ∼15%, SA2 and Pds5B (4PJU and 5HDT, respectively) are similar, with a TM-score of 0.44 (scores lower than 0.3 are spurious and alignment significance increases rapidly above 0.5). The top scoring local sequence alignment between the two HMM profiles was between Pds5B residues 311–418 and SA2 residues 285–400. Using TMalign to perform a pairwise structural alignment between these resulted in a fit with a TM-score of 0.73. The alignment is disrupted by a large indel in Pds5B — realigning SA2 to the region directly after this produces an improved TM-score of 0.64. The amino-terminal region of Pds5B has been truncated for clarity. (D) In the LECA Smc–kleisin ancestor, the Kite dimer was presumably flanked by the ancestral HEAT-protein/Hawk. Successive duplications of Hawks led to the Kites being displaced. The lack of Hawks in Smc5-6 suggests that it diverged earlier from the cohesins and condensins, whose specialised functions were facilitated by the recruitment of the Hawk family.

HEAT repeat proteins are a highly diverse family, a small subset of which regulate cohesin and condensins in eukaryotes (Figure 1A). Building on the recent description of the Kite family, we asked whether this subset descends from a common ancestral HEAT repeat protein. However, this question presents significant technical difficulties. Repetitive sequences can diverge rapidly; indeed, the average sequence identity between mammalian HEAT repeat proteins and insect orthologues is just ∼13% [3]. This makes accurate sequence alignment challenging, and classical methods for homology detection fail on all but the most similar of these proteins. To tackle this problem we developed a novel network-based approach. Briefly, this utilises extensive profile–profile HMM searches [4] to generate a network, which is then clustered to reveal groups of paralogous proteins (for details, see Supplemental Methods and Figure S1A in Supplemental Information, published with this article online).

Applying this method to budding yeast, we find that amongst a large number of diverse clusters, all HEAT repeat proteins known to interact with α-, β- and γ- kleisins form a distinct cluster (Figure 1B). Similarly, in humans, two closely interacting clusters containing all condensin and cohesin Hawks are formed (Figure S1B,C and Figure S2A). These clusters are robust to changes in network parameters and are highly significant (p-value < 1 x 10^-6^, permutation tests). Additionally, several other known protein families were recapitulated in individual clusters. GO-term analysis of biological processes also demonstrated highly significant enrichment in multiple clusters, *e.g.* the karyopherin α subunits, KPNA1–7. We therefore conclude that our method is effective and that Hawks form a distinct subgroup within the larger HEAT family.

Two important conclusions stem from these observations. First, NIPBL/Scc2 — the cohesin DNA loader — is confidently included in the Hawk cluster. This conclusion is now strongly supported by the recent biochemical studies of the *Chaetomium thermophilum* yeast Scc2, which is found to bind robustly to *C. thermophilum* Scc1 [5]. Second, our analysis fails to support the previous proposal that Nse5 and Nse6 associated with the eukaryotic Smc5-6 holocomplex contain HEAT repeats. Neither contains detectable repeats and searches for paralogues returned few proteins, none of which contained HEATs (see Supplemental Methods). Furthermore, a literature search revealed no evidence for Nse5 containing repeats, while the Nse6 annotation is based on a structural prediction which we were unable to replicate [6]. These negative findings indicate that Smc5-6 is alone amongst eukaryotic Smc–kleisin complexes in retaining Kites (the Nse1–3 subunits) and lacking Hawks.

We next turn to a possible origin for the Hawk family. Orthologues were found in almost all eukaryote species we tested, collectively accounting for all major extant branches of the eukaryotic tree. We searched for related sequences in Lokiarchaeota, currently the closest known archaeal relatives of the Last Eukaryotic Common Ancestor (LECA). Several lokiarchaeal HEAT repeat proteins produced significant alignments with Hawks; furthermore, we find that the lokiarchaeal HEATs predominantly cluster with clathrin adaptor proteins, which share sequence and structural similarity with the Hawks (Figure 1B and Figure S2B) [7]) These observations lead us to tentatively suggest that the ancestral Hawk protein derived from an ancient group of HEAT proteins related to the clathrin adaptor family, and that this occurred close to or even prior to the prokaryote–eukaryote split.

An independent test of our conclusion that Hawks derive from a common ancestor is provided by structural analysis of yeast subunits Pds5/Pds5B and Scc3/SA2 (*e.g.* 8, 9). From sequence analysis we see that the remaining Hawks are of a similar size, with similar distributions of repeats identified from sequence, particularly when compared to the clathrin adaptors (Figure S2C). Pds5B and SA2 also align well structurally, though disrupted by an indel in Pds5B (Figure 1C and Figure S2D) [10]. When this region was omitted, the alignment improved considerably (Figure 1C). Finally, SA2 and Pds5B display similar patterns of conservation along their spines (Figure S2E). These similarities between SA2 and Pds5B are particularly striking since SA2/Scc3 appears to be the most diverged member of the Hawk clusters. Supporting this further, the structure of Scc2 from *C. thermophilum* [5] shows that its carboxy-terminal region has a very similar shape and structure to Pds5, albeit lacking the indel found in the latter.

Based on our main conclusions — the strong clustering of Hawks, their deep conservation across eukaryotes, and their absence from Smc5-6 complexes — we propose a model for the Smc-kleisin complex in LECA (Figure 1D). According to our hypothesis, the ancestral Hawk protein was recruited to the complex very early in eukaryotic history. Successive duplications of this protein displaced the Kites, leading to the predecessor of modern condensins, containing two Hawk regulators (similar to budding yeast’s Ycs4 and Ycg1), and to the cohesins, with three (Pds5, Scc3 and Scc2). An outstanding question from this model is whether or not Smc5-6 gained and then lost Hawks, or whether its lack of Hawks indicates that it forms a branch distinct from the cohesins and condensins. In any case, our results show that the Smc–kleisins can be separated into two groups — those containing Kites, and those containing Hawks; of these, the Hawk–Smc–kleisins appear to be uniquely eukaryotic. Both cohesin and condensin share the ability to organize loops of chromatin fibres around an axial core and cohesin may have later acquired the ability to hold sister chromatids together and to be cleaved by separase. A key question now emerging is whether the replacement of Kites by Hawks was mechanistically associated with the acquisition of these novel functions.

## Supplemental Information

Document S1. Two figures
