# Supplementary material for: Evolution of condensin and cohesin complexes driven by replacement of Kite by Hawk proteins
Source: Curr Biol. 2017 Jan 9;27(1):R17–8. doi: 10.1016/j.cub.2016.11.050 (PMC5228436; doi:10.1016/j.cub.2016.11.050)
Supplement: Document S1. Two figures [file mmc1.pdf]

Supplemental Information: Evolution of condensin and cohesin complexes driven by replacement of Kite by Hawk proteins

Jonathan N. Wells, Thomas G. Gligoris, Kim A. Nasmyth and Joseph A. Marsh

Supplemental Methods

Source code and associated data

All source code and data associated with the following methods and analyses is available online at https://github.com/jonwells90/smc_hawks

Construction of homology networks

Proteome fasta files for *S. cerevisiae*, *S. pombe* and *H. sapiens* were downloaded from the Uniprot reference proteomes databank (04.2016) [S1]. HHSuite v.3.0.0 was compiled from source (git commit 45c5d85ddf241576b4b069f628e377977238efb9) [S2,S3]. HHsuite databases were constructed as per the protocol described in the HH-suite manual (available at http://www.mpibpc.mpg.de/soeding or https://github.com/soedinglab/hh-suite), using the clustered uniprot20_2016_02 database. It should be noted that due to the fact that HHsuite databases are generated from large multiple sequence alignments for each protein, the resulting species databases are not independent. Orthologous proteins in each species will, by virtue of that fact, produce profile HMMs with significant overlap.

Seed sequences for putative members of the Hawk family were selected semi-arbitrarily for each species (see supp. data file 1). Each seed was searched against the uniprot20 database using hhblits [S2] (local alignment, two iterations). Predicted secondary structure was added to each MSA/profile HMM using Psipred [S4]. The resulting profile HMMs were then searched against the relevant species-specific database using hhsearch (local alignment, single iteration, no pre-filter) to generate a list of at most 500 putative paralogues from each seed. In turn, each one of these sequences was subjected to the same procedure, producing a large set of nodes and edges, with nodes representing proteins and edges representing alignments between them, weighted by the rank of the alignment.

The resulting graph was filtered by removing edges arising from alignments with a length of less than 100 columns (accounting for the length of ~2 HEAT repeats), an expect-value of greater than 0.01 (thus controlling the false-discovery rate) or a true positive probability of less than 15%. Edge weights were then normalised according to the following formula

$$f(r) = \frac{1}{1 + \frac{99(r - r_{min})}{r_{max} - r_{min}}}, 1 \leq r \leq 500$$

Such that the normalised rank $f(r)$ lies between 0.01 and 1.0, with 1.0 being the best possible mean rank and 0.01 the worst.

At this stage, each edge has a direction, pointing from the protein used as a query sequence to the returned paralogous protein. As such, a given pair of nodes can be connected by either one edge or two; the former only being possible if a protein only appeared in the second round of searches and was therefore not queried itself. In order to make the graph undirected, all nodes with a degree of less than 2 were discarded and the remaining edges between each pair of nodes combined and weighted by the geometric mean of normalised alignment ranks. Since the geometric mean is always lower than the arithmetic mean, this avoids giving too much weight to results from proteins with very few significant alignments.

Finally, clustering was carried out using the mcl algorithm with an inflation parameter I = 2.5 for all networks [S5]. Initial network construction and parameter setting was performed on a fully-labelled *S. cerevisiae* network, but *S. pombe* and *H. sapiens* replicates were performed on blinded graphs, with genes in each cluster only being revealed after all filtering and cluster parameters had been fixed. GO term enrichment analysis was carried out using the Cytoscape BiNGO app, with GO "Biological Process" annotations [S6]. P-values were generated using the hypergeometric test and corrected for false discovery rate using the Benjamini-Hochberg method [S6,S7].

Homology network permutation tests
Assuming a null hypothesis under which alignment ranks contain no information about the relative likelihood of two proteins being related, a single control network was constructed for each species. This was generated from the observed network by randomising the edge weights between each pair of nodes. This was achieved by pre-filtering alignments as usual, but randomly assigning ranks. These were then normalised and averaged as for the observed network. Each random network was then clustered and each cluster tested for membership of Hawk proteins; specifically we ask: does there exist a cluster in the random graph containing exclusively those proteins from the largest Hawk cluster in the observed graph? This process was repeated 10^6 times for each species, and the resulting p-value calculated as the number of times the complete Hawk cluster was seen, divided by the number of trials.

Searching for lokiarchaeota HEAT repeat sequences
13 Lokiarchaeota proteins containing HEAT repeats were downloaded from the Uniprot database; 9 on the basis of Uniprot sequence annotations and an additional 4 proteins, including 2 fragments, on the basis of HHsuite searches and manual inspection. These sequences were searched against our human HHsuite database, and the resulting human sequences searched back against the lokiarchaeota database. A sub-graph was built using the same parameters as for the main eukaryote networks, leaving exactly 10 archaeal proteins remaining after quality control. The resulting set of edges was concatenated onto the human network and re-clustered.

Mapping of repeat domain boundaries
Sequences from *S. cerevisiae* Hawks and clathrin adaptors were used to generate multiple sequence alignments with HHblits. Multiple sequence alignments were generated with the uniprot20_2016_02 database. These alignments were subsequently passed to the HHRepID web server (https://toolkit.tuebingen.mpg.de/hhrepid). The threshold p-value for assigning repeat domain families was kept at 0.01, and the threshold for suboptimal self-alignments was set to 0.1, also the default. The number of HHblits iterations was set to 0 since we had produced our own MSAs in the preceeding step. Repeat predictions were collected from the HHRepID results with alignment stringencies between 0.0 and 0.3, depending on which value produced highest confidence predictions.

Structural alignments and conservation mapping
Structures for human Pds5B and SA-2 were downloaded from the PDB (5HDT and 4PJU respectively, 28.04.2016) [S8]. Structures were aligned in PyMol using TM-align, both globally and locally by splitting SA-2 and Pds5B at residues L436 and Y462 respectively and realigning each half [S9,S10]. Conservation mapping was performed using multiple sequence alignments generated as follows: For Pds5B and SA-2, 1000 metazoan sequences for each were retrieved from the NCBI non-redundant sequence database using blastp, then clustered to 90% sequence identity with usearch [S11,S12]. The remaining sequences were then aligned in forward and reverse directions with MAFFT, MUSCLE and GlProbs, with a final composite MSA being generated with MergeAlign [S13–S16]. Finally, these were mapped onto the PDB structures in Chimera [S17].

Analysis of putative Nse5 and Nse6 HEATS
Specific searches for HEAT-containing Nse5 and Nse6 homologues were carried out with the same parameters as for the main network – hhblits with 2 iterations to generate profile HMMs, followed by hhsearch to find significant alignments in the three main species datasets. Kre29 was used in place of Nse6 for *S. cerevisiae,* and Slf2 for Human. Subsequent searches using hhblits/hhsearch were carried out with more iterations for the hhblits step – this increases sensitivity but at the cost of accuracy in determining relative rank of alignments. Additional searches were performed in a wider variety of species using the proteome datasets available on the HHSuite webserver. Next, HHRepID [S18] was used to try and detect repeats within Nse5-6 themselves (as opposed to HEAT containing homologues). As before human Slf2 was also checked, as was Kre29. Iterations ranging from 3-8 were used to generate the profile HMMs, thus spanning a wide range of sensitivities.

Finally, a literature search was performed to try and identify the published evidence for the Nse5-6 HEAT annotations. On the basis of evidence for HEATs in Nse6 presented by Perbernard et al., [S19], we unsuccessfully attempted to replicate their finding using the structural prediction server 3D-PSSM, which is now obsolete [S20]. Following this, we used Phyre2 [S21], which supersedes 3D-PSSM. The Nse6 sequence (Uniprot id - O13688) was input to the server using default settings on the webserver. This did not yield HEAT proteins, and we were unable to find published evidence for Nse5 containing HEATs.

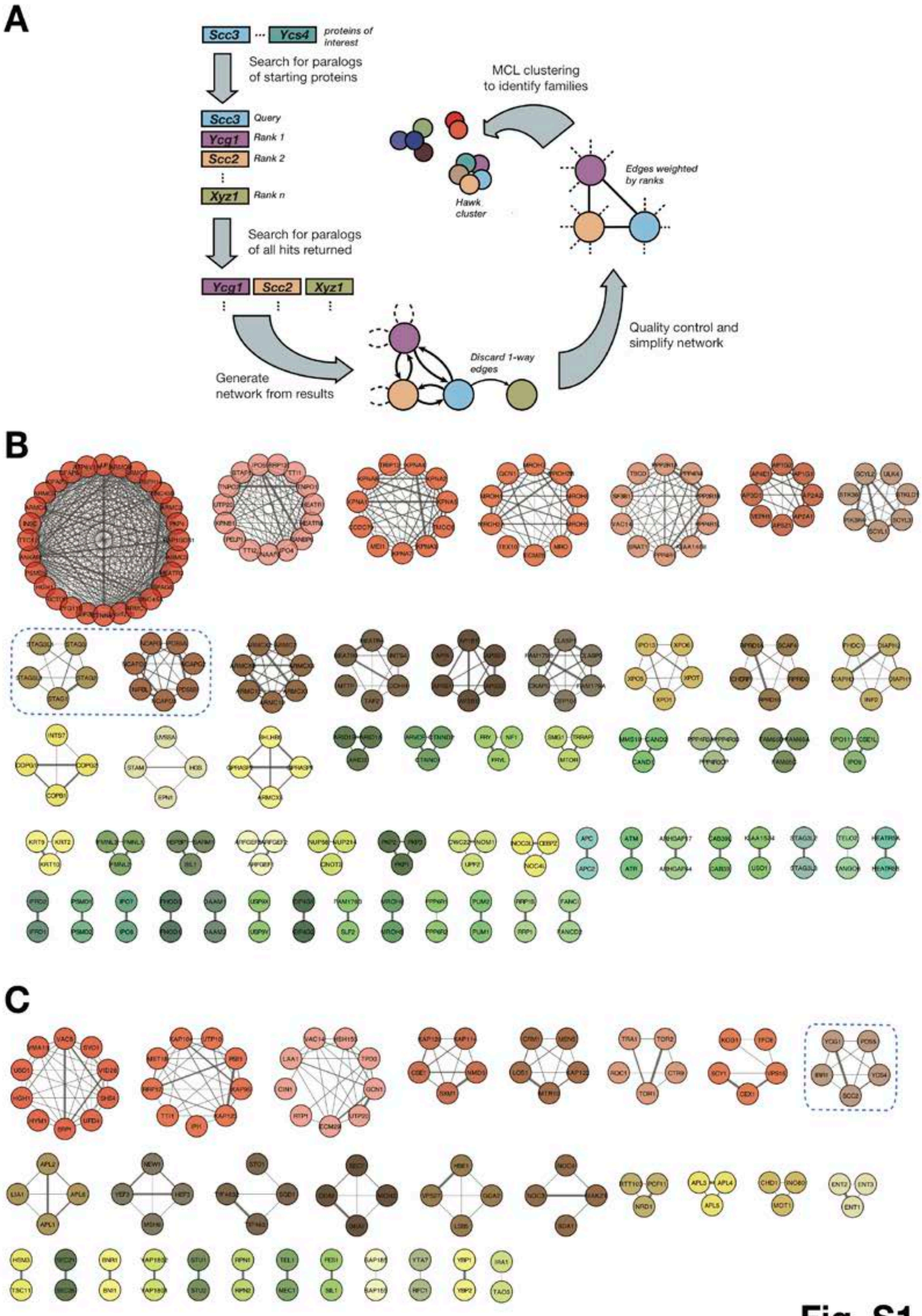
A
Scc3
Ycs4
proteins of interest
Search for paralogs of starting proteins
Scc3 Query
Ycg1 Rank 1
Scc2 Rank 2
Xyz1 Rank n
Search for paralogs of all hits returned
Ycg1
Scc2
Xyz1
Generate network from results
Discard 1-way edges
Quality control and simplify network
Edges weighted by ranks
MCL clustering to identify families
Hawk cluster
B
C

Fig. S1

Figure S1. Homology network construction workflow and raw clusters. Related to Figure 1B.
(A) We first select the set of proteins we are interested in. For each of these, we perform a search of the yeast database using hhblits and hhsearch to find paralogous proteins. The resulting set of hits is ranked according to the probability of the HMM profile alignment being a true positive (alignment of HMM profiles being the key feature that differentiates HHsuite from traditional pairwise alignment tools). Since a protein that has diverged significantly may produce spurious hits, we performed additional searches on all of the results; if protein A returns protein B as a hit, then we would expect to see A returned when searching for paralogues of B. If this is not the case, we ignore the relationship. Having performed searches on all of our proteins and putative paralogues, we then combine the data to produce a network. Each pair of nodes (proteins) is connected by two edges (alignments), each weighted by the rank of the alignment. After quality control, the network is simplified, with each pair of edges being converted to a single edge, weighted by the mean of the two edge weights. After final quality control, we then cluster the network using the MCL algorithm. Clusters generated from applying MCL algorithm to raw homology networks from (B) *Homo sapiens* and (C) *Saccharomyces cerevisiae*. Clusters containing Hawk family members are circled, intra-cluster edges have been hidden for clarity, and lines are weighted according to alignment rank (thicker lines correspond to higher average alignment ranks).

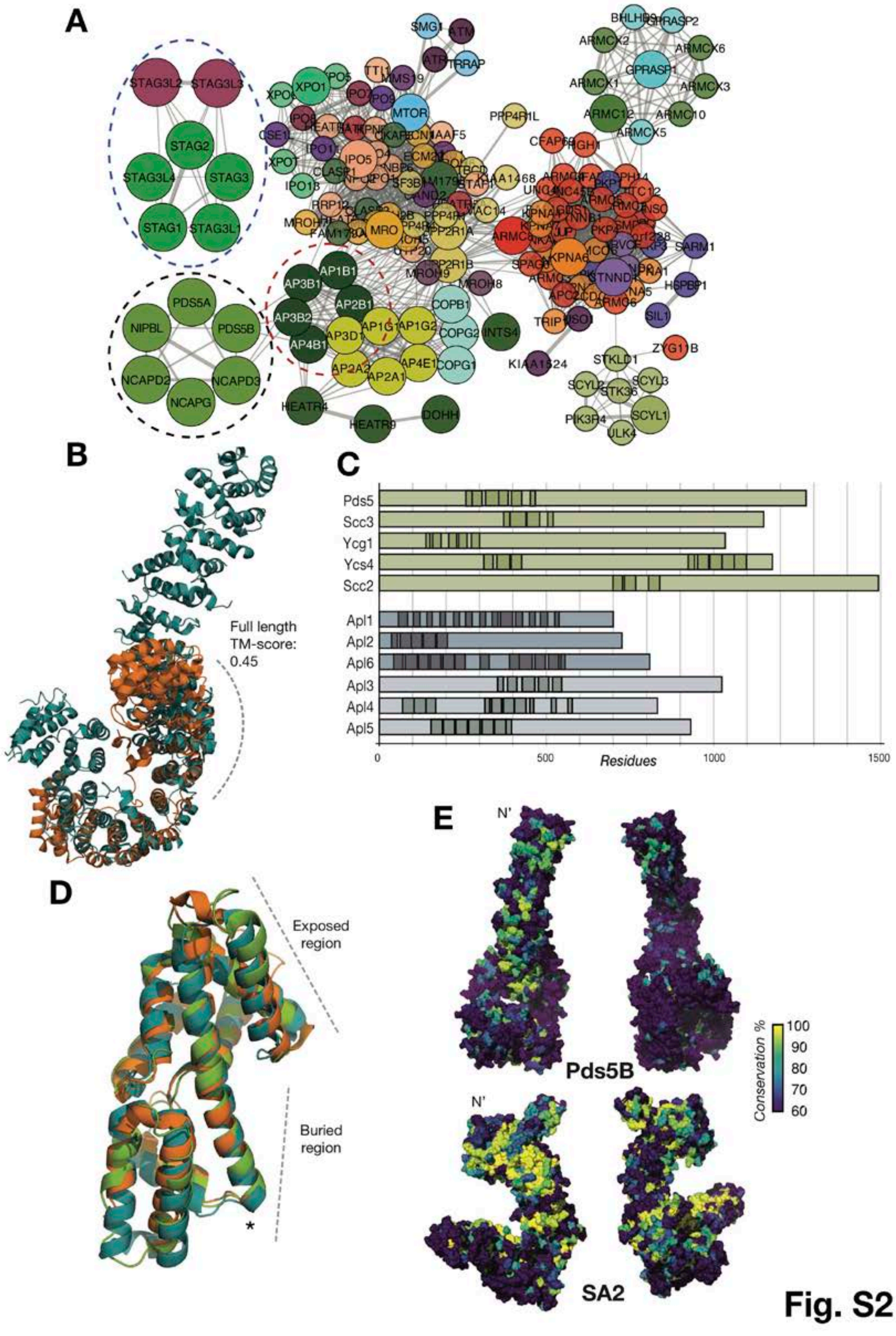
A
STAG3L2
STAG3L3
STAG2
STAG3L4
STAG3
STAG1
STAG3L1
PDS5A
NIPBL
PDS5B
NCAPD2
NCAPD3
NCAPG
AP1B1
AP3B1
AP2B1
AP3B2
AP4B1
AP1G1
AP1G2
AP3D1
AP2A2
AP2A1
AP4E1
COPB1
COPG2
COPG1
INTS4
HEATR4
HEATR9
DOHH
MTOR
XPO1
SMG1
ATM
TRRAP
MRO
PPP4R1L
ARMCX2
ARMCX6
ARMCX1
GPRASP1
ARMCX3
ARMC12
ARMC10
ARMCX5
GPRASP2
SARM1
HSPBP1
SIL1
ZYG11B
STKLD1
SCYL2
SCYL3
STK36
SCYL1
PIK3R4
ULK4
KIAA1524
KPNA6
STNND1
B
Full length
TM-score:
0.45
C
Pds5
Scc3
Ycg1
Ycs4
Scc2
Apl1
Apl2
Apl6
Apl3
Apl4
Apl5
0
500
1000
1500
Residues
D
Exposed
region
Buried
region
*
E
N'
Pds5B
N'
SA2
Conservation %
100
90
80
70
60

Fig. S2

Figure S2. Human homology network and additional structural analyses. Related to Figure 1. (A) Due to the larger size of the human network, only those edges with hhsearch true positive probability greater than 99.5% are shown. Cluster colours edited to correspond with Figure 1C. Orthologous (or partially orthologous) clusters are in identical colours to ones in *S. cerevisiae*. It should be noted that whilst SA-2 and close paralogues (blue circle) cluster separately from the rest of the hawks (black circle) - this is likely due to increased divergence amongst SA/Scc3 proteins. Members of the main hawk cluster are still amongst the top ranked proteins of SA group members, with highly significant alignments. The closely related Clathrin adaptor proteins are circled in red. (B) The Clathrin adaptors are a highly conserved family of proteins that share distant sequence homology with the Hawks. Here we show structural similarities between Human AP2B (2XA7, orange) and *L. thermotolerans* Pds5 (5F0O, teal) proteins. Pds5 aligns along the full length of AP2B with a TM-score of 0.45 – this is significantly above expected for unrelated folds, but nonetheless still implies significant differences. Whilst similar in gross morphology, care should be taken not to over-interpret short regions of good alignment, as these can be complicated by the presence of multiple highly similar HEAT repeats. (C) The Hawks are typically larger than the clathrin adaptor proteins, with repeats that are only weakly conserved at the sequence level. These are defined most clearly in the regions either side of the central Scc1 binding cleft (confirmed in published structures for Pds5 and Scc3). In contrast, the clathrin adaptors are shorter, with better-defined repeats that are detectable across larger regions of the sequences. All repeats were detected using HHRepID, with a self-alignment threshold p-value of 0.1. Protein lengths were calculated from Uniprot fasta sequences used to generate the repeats. Note that Scc2 contains a disordered N-terminal domain that varies dramatically in length across species, accounting for its larger overall size. (D) Structural alignment of the indel region from Pds5/B in *H. sapiens* (teal), *S. cerevisiae* (green) and *L. thermotolerans* (orange, 5HDT, 5FRR and 5F0N respectively). Whilst there is no clear sequence conservation, the extended alpha-helix (marked with asterisk) is apparently a defining feature of the region. (E) Metazoan orthologues of Pds5b (5HDT) and SA2 (4PJU) were retrieved using blastp, aligned and mapped onto the structures. Both proteins bind Wapl and Scc1, with conserved binding patches for both being limited to the front, convex faces of Pds5B and SA2 (left side of panel). Wapl binds near the N-terminus on both proteins, with Scc1 apparently binding along a broad region along the spines. In contrast, the rear, concave faces (right side of panel) are significantly less conserved.

Supplemental references

[S1] Consortium U. UniProt: a hub for protein information. Nucleic Acids Res 2015;43:D204–12.

[S2] Remmert M, Biegert A, Hauser A, Söding J. HHblits: lightning-fast iterative protein sequence searching by HMM-HMM alignment. Nat Methods 2011;9:173–5.

[S3] Söding J. Protein homology detection by HMM-HMM comparison. Bioinformatics 2005;21:951–60.

[S4] Jones DT. Protein secondary structure prediction based on position-specific scoring matrices. J Mol Biol 1999;292:195–202.

[S5] Van Dongen S. A Cluster algorithm for graphs. Rep - Inf Syst n.d.:1–40.

[S6] Maere S, Heymans K, Kuiper M. BiNGO: a Cytoscape plugin to assess overrepresentation of gene ontology categories in biological networks. Bioinformatics 2005;21:3448–9.

[S7] Benjamini Y, Hochberg Y. Controlling the False Discovery Rate: A Practical and Powerful Approach to Multiple Testing. J R Stat Soc Ser B 1995;57:289–300.

[S8] Berman HM, Westbrook J, Feng Z, Gilliland G, Bhat TN, Weissig H, et al. The Protein Data Bank. Nucleic Acids Res 2000;28:235–42.

[S9] Zhang Y, Skolnick J. TM-align: A protein structure alignment algorithm based on the TM-score. Nucleic Acids Res 2005;33:2302–9.

[S10] The PyMOL Molecular Graphics System, Version 1.8 Schrodinger, LLC. n.d.

[S11] Altschul SF, Gish W, Miller W, Myers EW, Lipman DJ. Basic local alignment search tool. J Mol Biol 1990;215:403–10.

[S12] Edgar RC. Search and clustering orders of magnitude faster than BLAST. Bioinformatics 2010;26:2460–1.

[S13] Katoh K, Misawa K, Kuma K, Miyata T. MAFFT: a novel method for rapid multiple sequence alignment based on fast Fourier transform. Nucleic Acids Res 2002;30:3059–66.

[S14] Edgar RC. MUSCLE: multiple sequence alignment with high accuracy and high throughput. Nucleic Acids Res 2004;32:1792–7.

[S15] Ye Y, Cheung DW, Wang Y, Yiu S-M, Zhan Q, Lam T-W, et al. GLProbs: Aligning Multiple

Sequences Adaptively. IEEE/ACM Trans Comput Biol Bioinforma 2015;12:67–78.
[S16] Collingridge PW, Kelly S. MergeAlign: improving multiple sequence alignment performance by dynamic reconstruction of consensus multiple sequence alignments. BMC Bioinformatics 2012;13:117.
[S17] Pettersen EF, Goddard TD, Huang CC, Couch GS, Greenblatt DM, Meng EC, et al. UCSF Chimera--a visualization system for exploratory research and analysis. J Comput Chem 2004;25:1605–12.
[S18] Biegert A, Soding J. De novo identification of highly diverged protein repeats by probabilistic consistency. Bioinformatics 2008;24:807–14.
[S19] Pebernard S, Wohlschlegel J, McDonald WH, Yates JR, Boddy MN, Yates 3rd JR, et al. The Nse5-Nse6 dimer mediates DNA repair roles of the Smc5-Smc6 complex. Mol Cell Biol 2006;26:1617–30.
[S20] Kelley LA, MacCallum RM, Sternberg MJE. Enhanced genome annotation using structural profiles in the program 3D-PSSM1. J Mol Biol 2000;299:501–22.
[S21] Kelley LA, Mezulis S, Yates CM, Wass MN, Sternberg MJE. The Phyre2 web portal for protein modeling, prediction and analysis. Nat Protoc 2015;10:845–58.
[S22] Branzei D, Sollier J, Liberi G, Zhao X, Maeda D, Seki M, et al. Ubc9- and Mms21-Mediated Sumoylation Counteracts Recombinogenic Events at Damaged Replication Forks. Cell 2006;127:509–22.
[S23] Kliszczak M, Stephan AK, Flanagan AM, Morrison CG. SUMO ligase activity of vertebrate Mms21/Nse2 is required for efficient DNA repair but not for Smc5/6 complex stability. DNA Repair (Amst) 2012;11:799–810.
[S24] Bermúdez-López M, Pociño-Merino I, Sánchez H, Bueno A, Guasch C, Almedawar S, et al. ATPase-Dependent Control of the Mms21 SUMO Ligase during DNA Repair. PLOS Biol 2015;13:e1002089.
[S25] Rowland BD, Roig MB, Nishino T, Kurze A, Uluocak P, Mishra A, et al. Building Sister Chromatid Cohesion: Smc3 Acetylation Counteracts an Antiestablishment Activity. Mol Cell 2009;33:763–74.
[S26] Chan KL, Roig MB, Hu B, Beckouët F, Metson J, Nasmyth K. Cohesin's DNA exit gate is distinct from its entrance gate and is regulated by acetylation. Cell 2012;150:961–74.
[S27] Ouyang Z, Zheng G, Tomchick DR, Luo X, Yu H. Structural Basis and IP6 Requirement for Pds5-Dependent Cohesin Dynamics. Mol Cell 2016:1–12.
[S28] Muir KW, Kschonsak M, Li Y, Metz J, Haering CH, Panne D. Structure of the Pds5-Scc1 Complex and Implications for Cohesin Function. Cell Rep 2016:1–11.
